# Phylogenetic Analysis of Allotetraploid Species Using Polarized Genomic Sequences

**DOI:** 10.1093/sysbio/syad009

**Published:** 2023-03-18

**Authors:** J Luis Leal, Pascal Milesi, Jarkko Salojärvi, Martin Lascoux

**Affiliations:** Plant Ecology and Evolution, Department of Ecology and Genetics, Uppsala University, Norbyvägen 18D, 75236 Uppsala, Sweden; Plant Ecology and Evolution, Department of Ecology and Genetics, Uppsala University, Norbyvägen 18D, 75236 Uppsala, Sweden; Science for Life Laboratory (SciLifeLab), Uppsala University, 75237 Uppsala, Sweden; Organismal and Evolutionary Biology Research Program, Faculty of Biological and Environmental Sciences, and Viikki Plant Science Centre, University of Helsinki, P.O. Box 65 (Viikinkaari 1), 00014 Helsinki, Finland; School of Biological Sciences, Nanyang Technological University, 60 Nanyang Drive, Singapore 637551, Singapore; Plant Ecology and Evolution, Department of Ecology and Genetics, Uppsala University, Norbyvägen 18D, 75236 Uppsala, Sweden; Science for Life Laboratory (SciLifeLab), Uppsala University, 75237 Uppsala, Sweden

## Abstract

Phylogenetic analysis of polyploid hybrid species has long posed a formidable challenge as it requires the ability to distinguish between alleles of different ancestral origins in order to disentangle their individual evolutionary history. This problem has been previously addressed by conceiving phylogenies as reticulate networks, using a two-step phasing strategy that first identifies and segregates homoeologous loci and then, during a second phasing step, assigns each gene copy to one of the subgenomes of an allopolyploid species. Here, we propose an alternative approach, one that preserves the core idea behind phasing—to produce separate nucleotide sequences that capture the reticulate evolutionary history of a polyploid—while vastly simplifying its implementation by reducing a complex multistage procedure to a single phasing step. While most current methods used for phylogenetic reconstruction of polyploid species require sequencing reads to be pre-phased using experimental or computational methods—usually an expensive, complex, and/or time-consuming endeavor—phasing executed using our algorithm is performed directly on the multiple-sequence alignment (MSA), a key change that allows for the simultaneous segregation and sorting of gene copies. We introduce the concept of genomic polarization that, when applied to an allopolyploid species, produces nucleotide sequences that capture the fraction of a polyploid genome that deviates from that of a reference sequence, usually one of the other species present in the MSA. We show that if the reference sequence is one of the parental species, the polarized polyploid sequence has a close resemblance (high pairwise sequence identity) to the second parental species. This knowledge is harnessed to build a new heuristic algorithm where, by replacing the allopolyploid genomic sequence in the MSA by its polarized version, it is possible to identify the phylogenetic position of the polyploid’s ancestral parents in an iterative process. The proposed methodology can be used with long-read and short-read high-throughput sequencing data and requires only one representative individual for each species to be included in the phylogenetic analysis. In its current form, it can be used in the analysis of phylogenies containing tetraploid and diploid species. We test the newly developed method extensively using simulated data in order to evaluate its accuracy. We show empirically that the use of polarized genomic sequences allows for the correct identification of both parental species of an allotetraploid with up to 97% certainty in phylogenies with moderate levels of incomplete lineage sorting (ILS) and 87% in phylogenies containing high levels of ILS. We then apply the polarization protocol to reconstruct the reticulate histories of *Arabidopsis kamchatica* and *Arabidopsis suecica*, two allopolyploids whose ancestry has been well documented. [Allopolyploidy; *Arabidopsis*; genomic polarization; homoeologs; incomplete lineage sorting; phasing; polyploid phylogenetics; reticulate evolution.]

Polyploidization, a major structural rearrangement that involves the addition of a complete set of chromosomes to the genome, is widely perceived as an important evolutionary force behind functional innovation, local adaptation, range expansion, and speciation (Adams and Wendel 2005; Otto 2007; Rieseberg and Willis 2007; Albertin and Marullo 2012; Beest et al. 2012; Van de Peer et al. 2017; Clark and Donoghue 2018; Li et al. 2018). Polyploid organisms attain a new set of chromosomes either through whole-genome duplication (WGD) arising within a single species (autopolyploids) or as a consequence of interspecific hybridization accompanied by doubling of homoeologous genomes (allopolyploids).

Understanding how the expansion of gene families brought about by polyploidization can lead to functional innovation and diversification is one of the main research questions in evolutionary molecular biology (Wolfe and Shields 1997; Bowers et al. 2003; Kellis et al. 2004; De Bodt et al. 2005; Dehal and Boore 2005; Aury et al. 2006; Braasch and Postlethwait 2012; Amborella Genome Project 2013; Macqueen and Johnston 2014; Schwager et al. 2017; Van de Peer et al. 2017; Li et al. 2018; Cerca et al. 2021). When performing phylogenetic inference of gene families or taxonomic lineages, however, attempts to clarify evolutionary relationships are often hindered by the very presence of species of hybrid origin. Phylogenetic reconstruction methods and tools based on the classic bifurcating tree model of evolutionary descent are liable to yield incorrect tree topologies when applied to taxonomic groups containing interspecific homoploid hybrid species or when there are significant levels of gene flow across relatively distant lineages due to introgressive hybridization (Linder and Rieseberg 2004; Dasmahapatra et al. 2012; Keller et al. 2013; Fontaine et al. 2015; Lamichhaney et al. 2015; Mallet et al. 2016). Likewise, failure to explicitly consider the reticulate origin of allopolyploids during phylogenetic inference can lead to the formulation of inaccurate evolutionary hypotheses (Oxelman et al. 2017; Rothfels 2021). Hence, alternative methodologies have been developed where hybridization and polyploidization are accounted for during tree inference by conceptualizing phylogenies as reticulate networks from the outset, as opposed to classic bifurcating trees (Linder and Rieseberg 2004; Huson and Bryant 2006).

## Reticulate Networks and Polyploid Phylogenetics: Previous Work

The last 20 years have witnessed the development of several computational tools that use a network approach to quantify levels of homoploid hybridization between species or identify horizontal gene transfer events. Some, such as Neighbor-Net (Bryant and Moulton 2004), produce split graphs that display conflicting signals in the data. Alternatively, there are explicit network methods—such as PhyloNet (Than et al. 2008; Wen et al. 2018), TreeMix (Pickrell and Pritchard 2012), PhyloNetworks (Solís-Lemus et al. 2017), SpeciesNetwork/BEAST2 (Zhang et al. 2018a), and GeneRax (Morel et al. 2020)—that produce reticulate networks (rooted, directed, acyclic graphs) representing inferred evolutionary relationships. SplitsTree4 (Huson and Bryant 2006) can perform both types of analysis. Although initially developed for haploid and diploid data, some (e.g., PhyloNet) have been subsequently used or adapted to explore phylogenies containing polyploids, as discussed below.

In more recent years, a new generation of protocols has been developed with the explicit aim of incorporating polyploidization events in phylogenetic networks (reviewed in Oxelman et al. 2017; Freyman et al. 2021; Rothfels 2021). This has been done, for instance, by exploring phylogenetic incongruences between nuclear markers and maternally inherited plastids (Bertrand et al. 2015), by minimizing deep coalescences (modified PhyloNet [Oberprieler et al. 2017]; AllCoPol [Lautenschlager et al. 2020]; MPAllopp/PhyloNet [Yan et al. 2022]), or by employing a Bayesian framework for inference of reticulate networks under the multispecies coalescent (AlloppNET/BEAST [Jones et al. 2013; Jones 2017]; homologizer/RevBayes [Freyman et al. 2021]). The development of these methods has opened the door for the reconstruction of the reticulate history of allopolyploids of unknown ancestry, although at the cost of some important trade-offs and increased implementation complexity, as discussed next.

Reconstruction of phylogenies containing allopolyploids usually involves two distinct phasing steps (Rothfels 2021). First, *haplotype phasing* is performed separately for each locus in order to segregate alleles located in different chromosomes (or subgenomes). At this stage, it is often possible to tell homoeologous gene copies apart, but they are yet to be assigned to their respective subgenomes. During the second phasing step, homoeologous gene copies are assigned to one of the two subgenomes, that is, classified according to their evolutionary history, a sorting process sometimes referred to as *phasing of gene copies* (Freyman et al. 2021).

For the most part, current protocols used in the reconstruction of polyploid phylogenies perform only the last phasing step (phasing of gene copies) and their usage often depends on the availability of genomic data for each polyploid that has already been haplotype-phased (in the form of multiple-sequence alignments (MSAs) or gene trees, one for each locus). Segregation of homoeologous gene copies is often accomplished using experimental methods. Experimental phasing has very high accuracy, but it requires either the sequencing of long DNA fragments (600–900 bp and above) or the use of specialized equipment and high levels of technical expertise (Browning and Browning 2011; Porubsky et al. 2017; Hu et al. 2021). Short-read sequencing (50–150 bp), currently the dominant type of DNA sequencing, produces read fragments with reduced polymorphic information and is therefore considered less suitable for experimental haplotype phasing as a standalone application (Browning and Browning 2011; Hu et al. 2021; Rothfels 2021). While short reads can be assembled into larger haplotypes or homoeologous sequences using phasing algorithms (Aguiar and Istrail 2012; Berger et al. 2014; Das and Vikalo 2015; Xie et al. 2016; Moeinzadeh et al. 2020), read-backed haplotype phasing can be computationally demanding when performed across long genomic segments (Motazedi et al. 2018; Moeinzadeh et al. 2020; Schrinner et al. 2020) and is generally considered less precise than experimental phasing, as the phase may be lost in regions where the haplotypes share long nonvariant sequences. Despite such hindrances, the widespread use of short-read sequencing, and consequent availability of genomic data for an ever-increasing number of species (Sayers et al. 2022), creates a strong incentive to perfect existing methods or develop new approaches suitable for inference of polyploid phylogenies based on short DNA fragments, and in particular for methodologies that could replace the current and rather complex multistep phasing process by a simpler and more efficient procedure.

## Main Contributions

In this article, we explore a simple but powerful new method for identifying the phylogenetic position of the parental species of an allotetraploid that requires just one single phasing step. The approach here described was developed specifically for short-read high-throughput sequencing (HTS) data and makes use of *polarized* nucleotide sequences when preparing the MSA prior to phylogenetic inference. Polarization in this context refers to the rules used to select the alleles when polymorphic genomic sequences based on HTS data are reduced to single base sequences, a collapsing process often performed before using standard off-the-shelf phylogenetic inference packages. When applied to an allotetraploid species, genomic polarization produces nucleotide sequences that capture the fraction of the polyploid genome that deviates from that of a reference sequence, usually one of the species included in the MSA. We show that if the reference sequence is one of the parental species, the polarized polyploid sequence has a close resemblance (high pairwise sequence identity) to the second parental species. This knowledge is harnessed to build a new heuristic method where, by replacing the allotetraploid genomic sequence in the MSA by its polarized version, it is possible to identify the phylogenetic position of the polyploid’s ancestral parents using an iterative process.

The proposed approach relies on several important innovations. First, phasing based on genomic polarization is a one-step procedure where the segregation and phasing of gene copies are performed simultaneously. Second, in our protocol, phasing is performed directly on the MSA, skipping altogether the need to use large read libraries (unlike standard haplotype phasing based on computational methods), a key change that speeds up the phasing process considerably. Finally, our protocol is built upon widely used pipelines developed to reconstruct phylogenies comprising only haploid and diploid species, with polarization constituting just one extra processing step executed after producing the MSAs and before carrying out phylogenetic inference.

Validation of the proposed method is first done using simulated data. We show empirically that phasing based on genomic polarization allows for the correct identification of both parental species of an allotetraploid with up to 97% certainty in phylogenies evolving in the presence of moderate levels of incomplete lineage sorting (ILS). We then apply the polarization protocol to reconstruct the *Arabidopsis* phylogeny, an extensively studied genus, in part because it includes *A. thaliana*, the foremost model species in plant biology. The *Arabidopsis* genus contains two well-known neo-allotetraploids: *A. kamchatica*, the result of hybridization between *A. lyrata* and *A. halleri* (Shimizu et al. 2005; Shimizu-Inatsugi et al. 2009; Schmickl et al. 2010), and *A. suecica*, which has *A. thaliana* and *A. arenosa* as parental species (Hylander 1957; Kamm et al. 1995; O’Kane et al. 1996; Jakobsson et al. 2006; Novikova et al. 2017).

## Materials and Methods

### Polarized Genomic Sequences

In modern molecular phylogenetics, evolutionary relationships between species, lineages, genes, or strains are inferred by tallying or modeling nucleotide or protein differences between homologous genomic sequences. In high-coverage, high-throughput genomic sequencing, detailed information can be obtained about structural variants present on each sequenced individual. Often—but not always (see Minh et al. 2020)—this information is partially discarded when preparing the MSA prior to carrying out the phylogenetic analysis: in the process of creating a single haploid representative sequence for each species (sometimes called the “consensus sequence”), heterozygous sites are collapsed by selecting one of the detected alleles (e.g., randomly or by using majority rule).

In this article, we describe a methodology where, instead of arbitrarily discarding information about variant sites, this knowledge is harnessed to reveal the parental species of an allopolyploid. A key difference between allopolyploids and diploid species is that while in the latter heterozygous sites represent the allelic diversity in a population, allelic richness in allopolyploids reflects also the presence of genomic differences between the two subgenomes, that is, between the two parental species. In fact, immediately after hybridization, the increase in allelic richness in the allopolyploid (vis-a-vis the source species) stems from differences in nucleotide composition between the two parental subgenomes. In principle, one way to identify the parental species would be to devise a method to produce two distinct consensus sequences for the allopolyploid, with each sequence being strongly biased (high pairwise sequence identity) toward one of the ancestral genomes.

**Figure 1.**
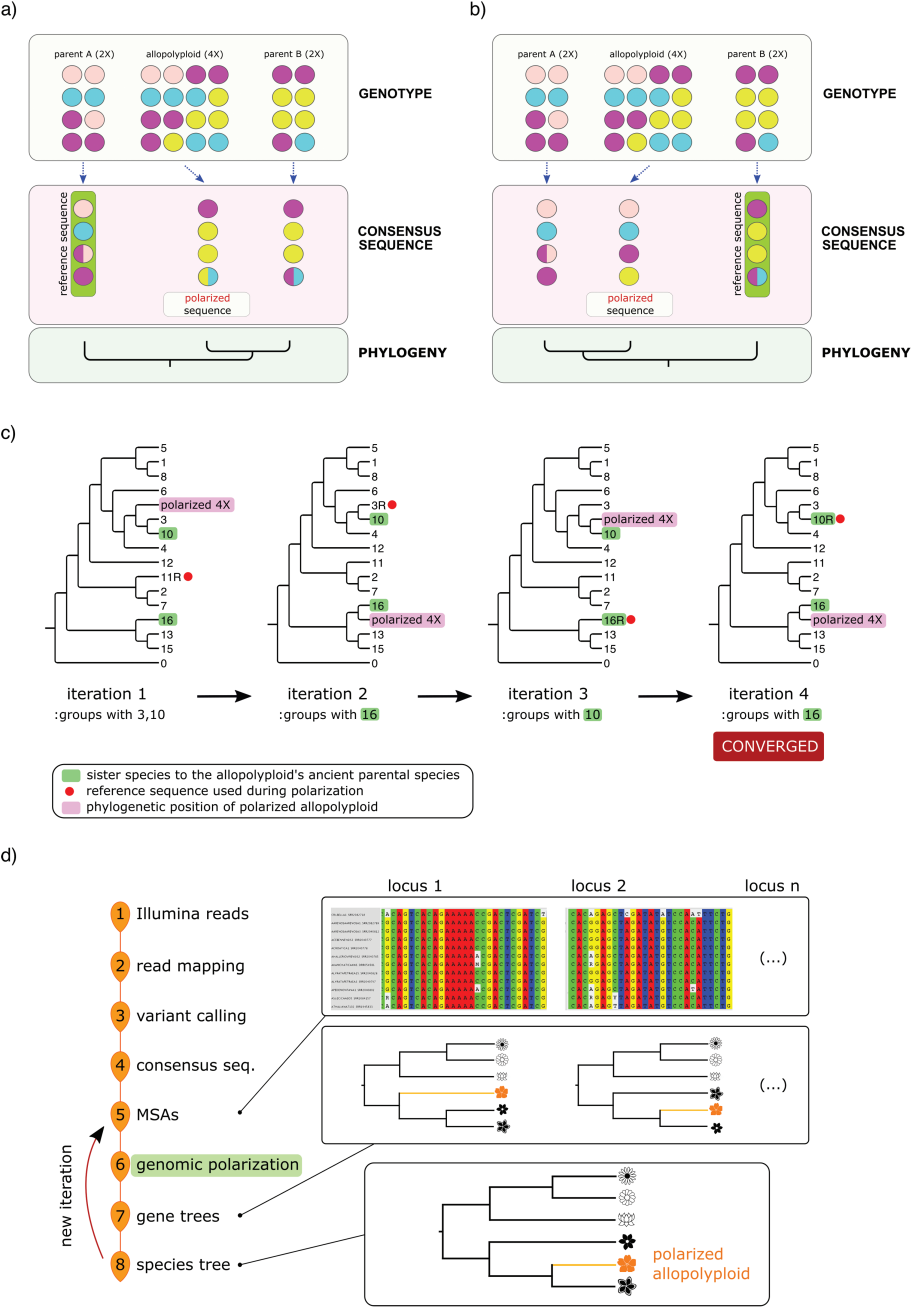
Iterative search and identification of an allopolyploid’s ancestral species. (a) Schematic diagram of the process used to create a polarized consensus sequence for an allotetraploid, when parent *A* is used as the reference sequence. The polarization process masks alleles in the allopolyploid that are also present in parent *A*, creating a sequence for the allopolyploid that closely resembles parent *B*. (b) Parent *B*, identified in the previous iteration, is now the reference sequence during polarization, leading to a polarized sequence for the polyploid that is phylogenetically closer to parent *A*. (c) Schematic representation of the phylogenetic position of the polarized allotetraploid sequence after each iteration, in a phylogeny containing sixteen species. Sequences “10” and “16” are the sister species to the allopolyploid’s parental species. Only the tetraploid sequence is polarized. Iteration 1: species “11,” selected randomly, is initially used as the reference sequence, and the polarized allopolyploid groups with species “3” and “10.” During the second iteration, one of species “3” or “10” is used as the new reference sequence (species “3” used in the present example) and the polarized polyploid groups with sequence “16.” From here on, the algorithm enters a bimodal equilibrium, with the polarized sequence alternatively grouping with sequences “10” or “16,” sister species to the tetraploid’s true ancient parental species. The location of the first parental species is found after two iterations (near species “16”), while convergence is attained by the fourth iteration (iteration 5 would have produced a result similar to that shown for iteration 3). Had species “16” (or “10”) been used as the initial reference sequence, convergence would have been attained after two iterations. (d) Synoptic diagram displaying the bioinformatics pipeline developed to identify the parental species of an allotetraploid using short-read data. See Materials and Methods for detailed description. MSA, multiple-sequence alignment.

When generating a consensus sequence, two fundamental aspects need to be considered: 1) the reference sequence to be used and 2) the criteria employed to select alleles at polymorphic sites. Let us assume that the reference sequence coincides with one of the parental species (“parent A”) and that, in the process of creating the consensus sequence for the allotetraploid, we give preference to alleles *not* present in the reference sequence (alternative-allele polarization), to a large extent masking the genomic signal created by subgenome-A (Fig. 1a). In this case, the (polarized) consensus sequence produced for the allopolyploid would be closer to “parent B,” the second parental species. Having identified “parent B” as a candidate parental sequence, the latter becomes the new reference sequence, and the polarization procedure is repeated anew, this time giving rise to a polarized consensus sequence for the allopolyploid that more closely resembles “parent A” (Fig. 1b).

In short, each polarized sequence captures one of the two main evolutionary histories present in the allopolyploid. The higher the number of genomic differences between the ancestral diploid lineages *prior* to the allopolyploidization event, an arrangement generally favored in allopolyploids, the stronger the contrast between the two polarized sequences. The average genetic divergence between progenitor species tends to be higher in allopolyploids than in interspecific homoploid hybrids (Chapman and Burke 2007; Paun et al. 2009), possibly because higher levels of genetic differentiation favor the formation of bivalent pairing of homoeologous chromosomes during meiosis in allopolyploids, reducing the incidence of meiotic abnormalities and improving fertility (reviewed in Mable 2004 and Paun et al. 2009). Post speciation, and as time goes by and the three species diverge, the parental signal is progressively masked in the allopolyploid due to structural rearrangements and new point mutations (Soltis and Soltis 1999; Dodsworth et al. 2016). However, there is evidence that subgenomes in recent allopolyploids often display a high degree of sequence identity and genomic synteny relative to the modern representatives of their respective progenitor species (Ainouche and Wendel 2014; Chen et al. 2020; Gordon et al. 2020; Burns et al. 2021; Schiavinato et al. 2021), and the conceptual model described above should therefore hold true.

Later in this article, we will use simulated data to show that this simple procedure allows us to pinpoint the position of the two parental species in a large phylogeny—independently of the species initially selected as the polarizing reference sequence. With each iteration, a new reference sequence is identified, with the process eventually converging toward a bimodal equilibrium state where the allopolyploid’s polarized sequence alternatively groups with one of the two species, among those included in the analysis, most closely related to the polyploid’s ancestral parents (Fig. 1c).

The proposed methodology is suitable for high-coverage short-read HTS data, such as whole-genome and -exome capture sequencing data commonly obtained using an Illumina platform. It can also be used with long-read sequencing data. The actual input data are made of hundreds or even thousands of individual MSAs, one for each locus, where each species included in the phylogeny, including polyploids, is represented by a single nucleic acid sequence (Fig. 1d). In other words, each species is represented by a single individual and, more importantly, the input data do not need to be pre-phased. Alignments can include full sequences (single-nucleotide polymorphisms [SNPs] together with invariant sites) or be composed exclusively of SNPs, although in the latter case it might then be necessary to apply an ascertainment bias correction model during phylogenetic inference (Lewis 2001; Leaché et al. 2015). Because genomic polarization is performed by comparing polymorphic sites across species, high-coverage sequencing data are recommended for the allopolyploid (>25×). This should yield about 1–2 million SNPs per species (cumulative across loci), which serve as the core information used in the polarization step and subsequent phylogenetic inference.

### Arabidopsis Libraries

Illumina whole-genome sequencing short-read data for 11 *Arabidopsis* accessions and 1 *Capsella rubella* (outgroup) library were downloaded from the European Nucleotide Archive (ENA, https://www.ebi.ac.uk/ena). Detailed information about each accession is provided in Supplementary Table S1, available on Dryad at https://doi.org/10.5061/dryad.dfn2z353j. The *A. thaliana* accession is part of the 1001 Genomes Project (Cao et al. 2011), while the remaining libraries are part of a recent study published by Novikova et al. (2016). This data set includes two allotetraploids (*A. suecica* and *A. kamchatica subsp. kamchatica*) and two autotetraploids (*A. arenosa ssp. arenosa* and *A. lyrata ssp. petraea*).

### Read Mapping and Variant Calling

The pipeline for read mapping and variant calling generally followed the GATK best practices workflow developed by the Broad Institute (DePristo et al. 2011; Poplin et al. 2018). Read mapping was performed with BWA-MEM v 0.7.17 (Li and Durbin 2009). All the *Arabidopsis* and *Capsella* libraries were mapped to the unmasked *A. lyrata* v2.1 reference genome (Hu et al. 2011; Rawat et al. 2015). Genotyping was performed with GATK v 3.8-0 (McKenna et al. 2010) followed by site-wise hard filtering of variants. Loci found to be nonvariant after genotyping were also included in the output VCF file. During genotyping, only information for variant and invariant sites located in nuclear single-copy orthogroups was retained (such genomic regions were specified by using the option --intervals while performing hard filtering with GATK). Information about sites located outside these genomic regions was discarded. The orthogroups were identified with OrthoFinder v 2.3.1 based on the *A. lyrata* primary-transcript proteome (Emms and Kelly 2015). The final number of variants retained, as well as the average read depth within the genes of interest, is shown in Supplementary Table S2 available on Dryad. Detailed description of the pipeline and parameters used during read mapping and variant calling is provided in Supplementary Materials.

Alignment against a single reference genome has been shown to sometimes impact different samples differently and to introduce biases in downstream analyses (Pool et al. 2010; Sousa and Hey 2013; Bertels et al. 2014; Gopalakrishnan et al. 2017; Hurgobin and Edwards 2017). We therefore ran a parallel pipeline where read mapping and variant calling were performed for all samples using the *A. halleri* v.2.2 assembly (Briskine et al. 2017) as the reference genome.

### Consensus Sequences

Consensus sequences for each accession were generated by applying variants specific to each individual (as listed in the VCF file) to the reference genome fasta file. We used bcftools v 1.12 (Danecek et al. 2021) to create consensus sequences for each sample (bcftools consensus), with heterozygotic sites coded according to IUPAC nomenclature (Cornish-Bowden 1985). While the VCF file lists both SNPs and indel variants for each sample, at this stage only invariant sites and SNPs were included in the consensus sequence, ensuring that the total base-pair length of each consensus sequence matches that of the reference genome and, more importantly, that the start/stop position of each gene remains unchanged. Failed variants, sites without coverage, and deletions (but not insertions) were subsequently masked (N) in each consensus sequence using maskfasta from bedtools v 2.29.2 (Quinlan and Hall 2010).

### Multiple-Sequence Alignment

Each consensus sequence (one for each species) was split into individual fasta files, one for each locus, using getfasta from bedtools, based on the gff3 annotation file associated with the reference genome. Only protein-coding regions within the gene set of interest were included (single-copy orthogroups, as described above). Next, the fasta sequences associated with the same gene, but generated for different accessions, were collected into a single multi-fasta file. Because all gene sequences were collected based on a common reference genome, they required no further alignment. Thus, the file created for each gene family mimics an MSA fasta file. Finally, columns with more than two masked sites were removed from the MSA by running trimAl v 1.4.1 (Capella-Gutiérrez et al. 2009) using the -gapthreshold option. In order to use this package to remove masked sites, masked sites were recoded prior to running trimAl (and recoded back afterwards).

Because the use of different genetic markers can lead to distinct tree topologies (Jarvis et al. 2014; Chen et al. 2017; Chan et al. 2020), we also ran a parallel pipeline with MSAs containing only intronic regions. Being less conserved, introns usually have a higher number of phylogenetically informative sites. However, their inclusion may lower the confidence in the degree of site orthology across taxa (Thomson et al. 2010). By performing a separate analysis for exonic and intronic regions, we can evaluate whether the same parental pair is identified despite potential changes in tree topology stemming from the choice of genomic data used.

### Polyploid Polarization

In the following, we use the expressions “reference sequence” or “polarizing sequence” to indicate the species used to polarize the allopolyploid sequence during the polarization procedure. The reference sequence can be any of the sequences present in the MSA, apart from the focal allopolyploid. It should not be confused with “reference genome,” which in this article is employed exclusively to refer to the species used during read mapping, variant calling, and generation of the consensus sequence for each species prior to building the MSA, as described above.

Before carrying out the phylogenetic inference, the consensus sequence for the allopolyploid was polarized by selecting alleles in heterozygous sites *not* present in the reference sequence (alternative-allele polarization). If the site is fixed in the allopolyploid, it remains so in the polarized sequence. If all the alleles observed in the reference sequence are also found in the polyploid and there are no other alleles present in the latter, the site remains unchanged (based on our experience, this was a rare occurrence). Sites masked either in the polyploid or in the reference sequence are masked in the polarized sequence as well. Like all other sequences in the MSA, the polarized polyploid sequence is recoded according to the IUPAC nomenclature (Cornish-Bowden 1985). Sequences associated with diploid and autopolyploid species included in the analysis do not undergo polarization. During the first iteration, the reference sequence is selected either randomly or by the user. Afterwards, the reference sequence is chosen based on the phylogenetic position of the polarized allopolyploid sequence as determined in the previous iteration (closest relative to polarized allopolyploid; if the latter groups with a sister clade containing more than one taxon, one of them is selected randomly). Link to scripts used to perform polarization of genomic sequences is provided at the end of the article.

### Phylogenetic Analysis

Phylogenetic inference was initially carried out separately for each gene family by maximum-likelihood with IQ-TREE2 v 2.0-rc2-omp-mpi (Minh et al. 2020) using 1000 ultrafast bootstrap replicates (Hoang et al. 2018), exhaustive tree search mode (--allnni), and additional optimization to reduce risk of overestimating branch support (-bnni). Substitution model selection was performed with the ModelFinder algorithm (Kalyaanamoorthy et al. 2017) as implemented in IQ-TREE2 (-m MFP). As alignments include both variant and invariant sites, no ascertainment bias correction is required (Lewis 2001; Leaché et al. 2015; IQ-TREE Manual, http://www.iqtree.org/doc/iqtree-doc.pdf, accessed 3 May 2021). The analysis was carried out independently for each allopolyploid species. Because model search was performed independently over thousands of gene families, there was no single best substitution model. For the *Brassicaceae* MSAs, the HKY+F+G4 model (*F*: empirical base frequencies; *G4*: discrete Gamma model with four rate categories) was the most common best-fit model based on the Bayesian information criterion. Individual MSAs with less than 100 parsimony-informative sites, containing over 50% of gaps or other ambiguities, comprising two or more identical nucleotide sequences, or for which IQ-TREE2 failed to converge, were discarded and subsequently omitted during downstream analysis.

Single-locus phylogenies obtained using the method described above were used to estimate a species tree using ASTRAL v 5.7.3 (Zhang et al. 2018b), a super-tree inference method statistically consistent under the multispecies coalescent model and therefore able to handle substantial levels of ILS. ASTRAL was run in exact mode (-x) using all gene trees that passed the filtering step described in the previous paragraph, usually between 500 and 3000 (exact values provided in figure captions and main text). As recommended by the authors (Zhang et al. 2018b), branches in gene trees with low bootstrap support (≤30%) were contracted before running ASTRAL.

When plotting the cladograms produced by ASTRAL, we opted for showing branch quartet support values (*q*_*1*_/*q*_*2*_/*q*_*3*_) instead of the default measure of branch support provided by ASTRAL, which the authors call “local posterior probabilities” (PP) (Zhang et al. 2018b). The ASTRAL algorithm first computes quartet scores and then estimates PP based on the normalized quartet frequencies and the number of genes used in the analysis (ASTRAL manual, https://github.com/smirarab/ASTRAL/blob/master/astral-tutorial.md, accessed 5 August 2021). If more than 500 genes are used, then even the tiniest of differences between *q*_*1*_ and *q*_*2*_ yields a PP close to 1 for the *q*_*1*_ topology (Sayyari and Mirarab 2016). In the analyses carried out in this article, data sets of up to several thousand genes were used, which invariably led to phylogenies where all branches had PP equal to 1. We found these PP values to be uninformative and opted instead for showing quartet support values, as we believe they provide a better description of the level of incongruence between gene trees.

### Simulated Data Sets

We used synthetic data to evaluate the accuracy of the polarization protocol described in this article, specifically its ability to correctly identify the parental species of an allopolyploid in a large phylogeny. SimPhy v 1.0.2 (Mallo et al. 2016), a phylogeny simulator that can be used to simulate the evolution of gene families under the multispecies coalescent model, was used to generate species trees and the associated individual gene trees evolving under ILS. We used simulation conditions similar to those suggested by Mirarab and Warnow (2015) and Zhang et al. (2018b) to explore four different scenarios that reflect distinct levels of ILS (moderate or high) and speciation landscapes (recent or ancient speciation events). One hundred independent replicates were simulated for each scenario, with each replicate based on a different species tree topology. The species tree is randomly generated but included always 16 species plus an outgroup, with two individuals per species (-si f:2). For each replicate, 1200 independent gene families were generated.

MSAs were generated for each gene family with INDELible v 1.03 (Fletcher and Yang 2009) based on the individual gene trees created in the previous step, with mean nucleotide sequence length and standard deviation set to 1500 and 150 bp, respectively. For each MSA, the two individuals belonging to the same species were used to create diploid individuals, and two species were used to create the allotetraploid. The identity of the tetraploid’s parental species and the initial reference sequence used during polarization were both selected randomly for each replicate. After removing the two parental species from each MSA, the nucleotide sequence associated to the tetraploid was polarized, and individual trees were inferred for each gene family with IQ-TREE2. Lastly, we used ASTRAL to infer the species-wide tree on the basis of the individual gene trees obtained in the previous step. This process was run iteratively until convergence. See Supplementary Materials for further details.

### “No-ILS, No-Polytomy” Simulated Data Set

A special synthetic data set was also generated that included only phylogenies that satisfied a strict set of conditions. These included absence of ILS and no short internal branches (quasi-polytomies). By removing the two most common issues bedeviling tree inference algorithms, it became possible to accurately evaluate the protocol’s intrinsic ability to solve a phylogenetic reticulation problem (allopolyploidization, in this case). The absence of short internal branches was enforced by equalizing branch lengths after tree generation. As before, parental species were removed from the MSA after the allopolyploid was created. Evaluation of the “no-ILS-no-polytomy” data set was performed using 100 replicates, each containing a different random generated tree containing 16 species and an outgroup, but in this particular case, each replicate contained one single gene family and the associated MSA had very long nucleotide sequences (fixed at 20,000 bp). For each replicate, we independently applied the polarization protocol to all possible parental pairs and, for each parental pair, all possible reference sequences. For each of the {parent-A, parent-B, reference} sets, we then evaluated how many iterations it took until convergence, and whether both parental species were correctly identified. For this particular dataset, we restricted the analysis to cases where each parental species has one single sister species (as opposed to having a sister clade containing two or more species), and excluded scenarios where the parental species are sister species or nested species (a situation that requires a tailored approach, as discussed later in the article).

## Results

### Simulated Data

Runs based on synthetic data explored five distinct scenarios, reflecting different levels of ILS—none, moderate, or high—and whether speciation events took place deep into the phylogeny or more recently. Data sets with either moderate or high levels of ILS were generated using the exact same conditions used previously by Mirarab and Warnow (2015) and Zhang et al. (2018b) to evaluate the super-tree inference method ASTRAL (see Supplementary Materials for details).

**Figure 2.**
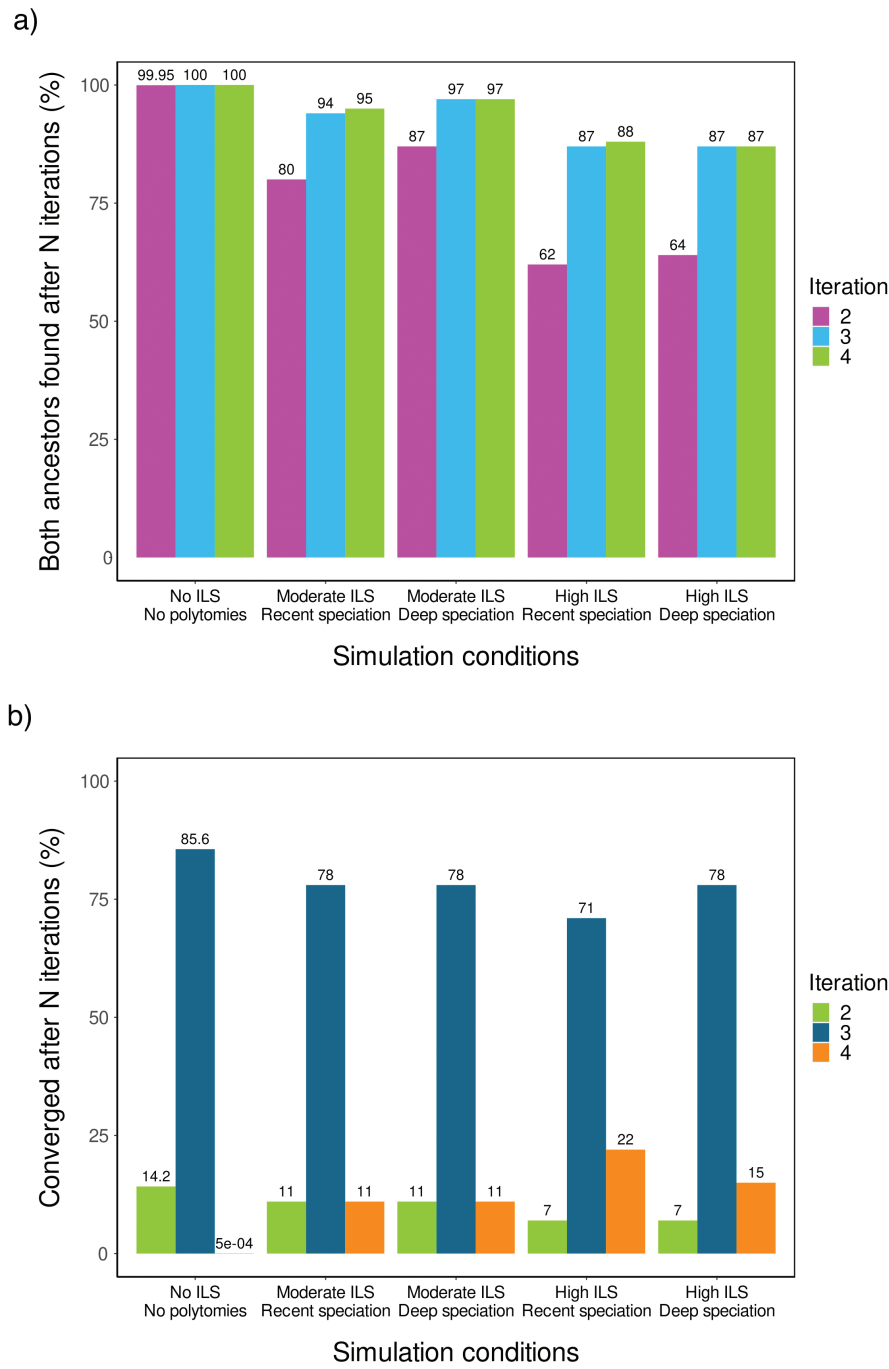
Evaluation of method accuracy using simulated data. (a) Percentage of replicates for which the phylogenetic position of both ancestral species of the allotetraploid were correctly identified, after each iteration and as a function of simulation conditions. (b) Percentage of replicates for which convergence was achieved, after each iteration and for different simulation conditions, independently of whether the two species found are the true parental species. One hundred independent replicates were run for each of the five ILS/speciation scenarios. ILS, incomplete lineage sorting.

We started by applying the polarization protocol to the dataset containing phylogenies with no ILS. This particular dataset also excluded quasi-polytomies, that is, phylogenies containing short internal branches. The main questions addressed through the analysis of the “no-ILS-no-polytomy” data set were, “Is the outcome of the polarization protocol affected by the choice of reference sequence?”, and “Does the protocol always converge towards the correct answer?” The “no-ILS-no-polytomy” data set included 100 replicates, each containing a different tree with 16 species and an outgroup. We tested all possible parental pairs for each tree and, for each parental pair, all possible reference sequences. Some parental pairs were excluded from the analysis based on topology (see Materials and Methods for details). Out of the 61,319 remaining permutations, the phylogenetic position of a true parental species was correctly identified in 61,290 cases (99.95%) during the first iteration (*N* = 1). In the remaining cases (29 of 61,319 [0.05%]), the position of the first parent was found during the subsequent iteration (*N* = 2). In all cases, polarization of the allopolyploid using the sister species of “Parent A” always correctly identified the phylogenetic position of “Parent B” and vice-versa. Correct identification of both parental species occurred in all cases studied upon convergence (100% rate of success; Fig. 2). We also checked the pairwise sequence identity between the polarized allopolyploid and each of the sequences present in the MSA. As expected, the polarized polyploid shows the highest pairwise sequence identity when compared with the sister species of one of the parental species (99.7% of all cases studied), no matter which species in the phylogeny is used as the polarizing (reference) sequence. These results show that, for this type of topologically constrained phylogenies, correct identification of both parental species of an allopolyploid is guaranteed (>99.998% ~61,319/61,320) and is independent of the species initially selected as the reference sequence.

Next, we explored synthetic phylogenies free of any topological constraints. Analysis of phylogenies based on simulated data revealed that the polarization protocol correctly identifies the position of both parents of an allopolyploid 94–97% of the time, after three iterations, if levels of ILS are moderate (Fig. 2a). Accuracy was somewhat lower (87%, after three iterations) when levels of ILS were high. A decrease in accuracy as levels of ILS are increased was expected, as it is known that the performance of ASTRAL, the species-tree inference method used during the analysis, is negatively impacted when processing datasets with high levels of gene discordance (Zhang et al. 2018b). Speciation time (more recent or far into the past) had no major impact on the protocol’s ability to correctly identify the allopolyploid’s ancestral species (Fig. 2a).

In every simulation carried out for phylogenies containing some level of ILS, at least one of the parental species was correctly identified. In the moderate ILS scenario, and for the few replicates in which the protocol failed to identify the position of the second parental species (4% of all replicates), the species trees almost always (9 times of 10) included one or more very short internal branches within the focal clade. In this particular set of cases, quartet support for the branch containing the allopolyploid suggested that there was more than one highly likely tree topology. In other words, the phylogenetic signal did not contain enough information to resolve the species tree in phylogenies containing very short internal branches and some degree of ILS.

In most cases, bimodal convergence was achieved after three iterations (Fig. 2b). In some instances (7–11% of all cases), convergence was attained after only two iterations. Very rapid convergence (*N* = 2) can be observed if, by chance, the reference sequence used during the first iteration is a sister species to one of the polyploid’s parental species. Longer convergence times (*N* > 3) were usually observed if it took more than one iteration to find the first parental species, an issue that can be exacerbated by the presence of high levels of ILS (Fig. 2b).

Although bimodal convergence was the norm, 10% of all simulation runs initially converged toward one single phylogenetic position. Unimodal convergence was observed in two distinct situations: when the polyploid’s parental species were nested (Supplementary Fig. S1a available on Dryad) or when the parental species are sister species (Supplementary Fig. S1b). Nested parental species describes the case where the sister clade to one of the parental species ({3, 11, 1, 4} in Supplementary Fig. S1a) includes the sister group to the second parental species ({4}). These two types of phylogenies (nested vs. sister parental species) can be disentangled by carrying out one extra iteration, but this time with the polarizing reference sequence being selected among those species furthest distant phylogenetically from the sole parental species identified so far (species “15” and “5” in Supplementary Figs. S1a,b, respectively, during iteration 3). If the parental species are nested, this procedure will pinpoint the location of the second parental species (Supplementary Fig. S1a). If the parental species are sister species, the position of the polarized allopolyploid remains unchanged (Supplementary Fig. S1b).

In summary, we started by using simulated data to identify the conditions under which the polarization protocol consistently yields the right solution, correctly establishing the identity of both parental species of an allopolyploid. We then proceeded to evaluate the protocol’s effectiveness when these conditions are violated. The protocol provides the correct solution (>99.998% accuracy) in phylogenies with no ILS, no internal short branches, and when the ancient parental species have one single extant sister species. Robust results were also observed in more realistic unconstrained phylogenies evolving in the presence of moderate levels of ILS (up to 97% accuracy), although the presence of quasi-polytomies in the focal clade can create uncertainty concerning the phylogenetic position of the parental species. Very high levels of ILS lead to a deterioration of the method’s ability to accurately identify both parental species (87% accuracy).

### Analysis of Biological Data Sets: Arabidopsis Genus

Having evaluated the accuracy of the polarization protocol with the help of synthetic data, we further tested its suitability for investigating the origins of recent allopolyploids by reconstructing the *Arabidopsis* genus, a group containing two autotetraploids, *A. lyrata* and *A. arenosa*, both of which originated through WGD from their respective diploid counterparts, as well as two neo-allotetraploids whose parental lineage has been well documented: *A. kamchatica*, the result of hybridization between diploid *A. lyrata* and diploid *A. halleri* (Shimizu et al. 2005; Shimizu-Inatsugi et al. 2009; Schmickl et al. 2010), and *A. suecica*, which has the diploid *A. thaliana* and the tetraploid *A. arenosa* as parental species (Hylander 1957; Kamm et al. 1995; O’Kane et al. 1996; Jakobsson et al. 2006; Novikova et al. 2017).

**Figure 3.**
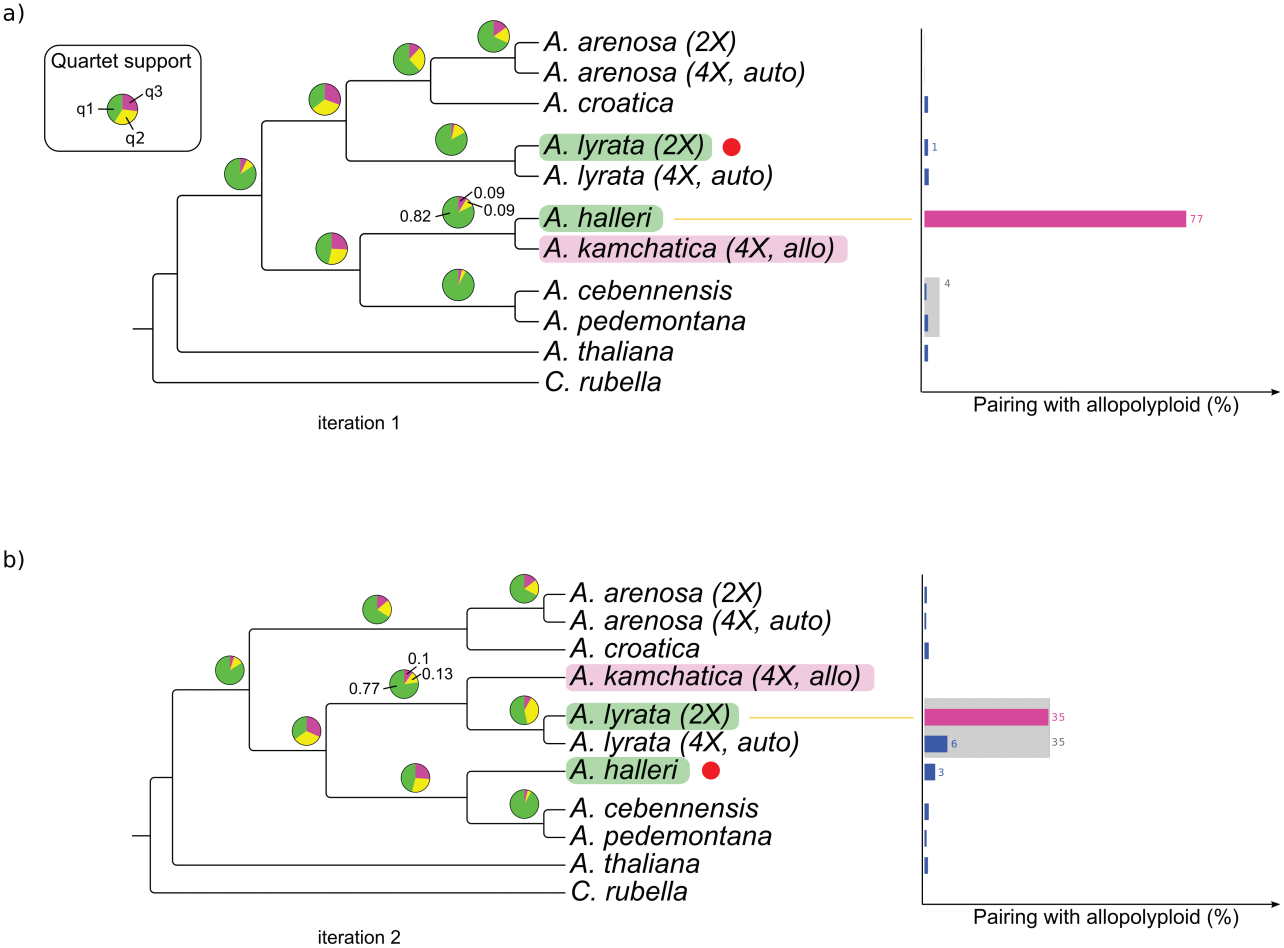
Phylogenetic origins of *A. kamchatica* confirmed using polarized exonic sequences. Species tree cladograms for the *Brassicaceae* genus, including the allotetraploid *A. kamchatica*, obtained with ASTRAL based on 905 (*N* = 1) to 816 (*N* = 2) gene families (exonic sequences only). *A. halleri* and *A. lyrata* were identified in previous studies as the parental species of *A. kamchatica*. (a) Species tree obtained after the first iteration (*N* = 1). *A. kamchatica* sequence polarized using *A. lyrata* as the reference sequence (red dot). *A. kamchatica* pairs with *A. halleri*. (b) Species tree obtained after the second iteration (*N* = 2). *A. halleri*, having been identified as a parental species during the previous iteration, is now the polarizing reference sequence. *A. kamchatica* pairs with *A. lyrata.* Pie charts show the quartet support for each branch. Precise support values are shown for the focal clade containing the allotetraploid species. Only the *A. kamchatica* sequence is polarized. Species containing diploid (2×) and autotetraploid (4×, auto) variants are labeled individually. Species with no label are diploid. Barplots on the right-hand side show frequency with which *A. kamchatica* pairs with other species and is based on phylogenetic analysis of individual gene families using IQ-TREE2. Blocks in gray indicate groupings with more than one species (shown only if support ≥4%, for clarity purposes).

We started by polarizing *A. kamchatica* using *A. lyrata* as the reference sequence (first iteration, *N* = 1). In the resulting species-wise phylogeny, there was strong quartet support (*q*_*1*_ = 0.82) for the grouping between *A. kamchatica* and *A. halleri* (Fig. 3a), in accordance with our expectations. Branches with a *q*_*1*_ value close to 1 are only observed if most individual gene trees are congruent for one specific topology. During the second iteration (*N* = 2), we used the putative parental species found in the previous iteration, *A. halleri*, as the polarizing sequence. This yielded a phylogeny (Fig. 3b) where *A. kamchatica* groups with the clade formed by the diploid and tetraploid *A. lyrata* sequences (*q*_1_ = 0.77).

These results, obtained using exonic sequences, were replicated using intronic sequences (Supplementary Fig. S2 available on Dryad). As reported in previous studies (Novikova et al. 2017), there is uncertainty concerning the position of the *arenosa–croatica* group. Likewise, in our analysis, it sometimes paired with the *A. lyrata* clade (Fig. 3), while some other times it was closer to the *halleri*–*cebennensis*–*pedemontana* group (Supplementary Fig. S2).

The cladograms shown in Fig. 3 are species super-trees estimated using ASTRAL, based on individual phylogenies inferred with IQ-TREE2 for 905 (*N* = 1) and 816 (*N* = 2) gene families (the number of gene trees can change from iteration to iteration, depending on the number of gene families that pass all filtering steps—see Materials and Methods for further details). The species tree provides insights on the origins of an allopolyploid, based on the available data. It renders a less clear image when it comes to identifying other species with which the allopolyploid may hybridize. In order to evaluate whether a third species may have contributed to the allopolyploid genome via, for instance, introgressive hybridization, we plotted the number of times the polarized allopolyploid species pairs with any of the other sequences included in the phylogeny, based on analysis of the tree inference results for individual gene families (IQ-TREE2 output). During the first iteration, pairing between *A. kamchatica and A. halleri* was observed in 77% of gene families (Fig. 3a, right panel). Groupings with other species or group of species were observed only in the low single digits, percentage-wise, and could be due to the presence of modest levels of ILS, weak phylogenetic signal, or noise (errors during sequencing, variant calling, or phylogenetic inference). During the second iteration, *A. kamchatica* grouped with diploid *A. lyrata* more frequently than with tetraploid *A. lyrata* (35% and 6%, respectively), and with both *A. lyrata* sequences 35% of the time (shown in gray). This is in agreement with the literature, as it has been suggested that the diploid variant of *A. lyrata* is the true ancestral taxon of *A. kamchatica* (together with *A. halleri*; Shimizu-Inatsugi et al. 2009; Koch 2019).

**Figure 4.**
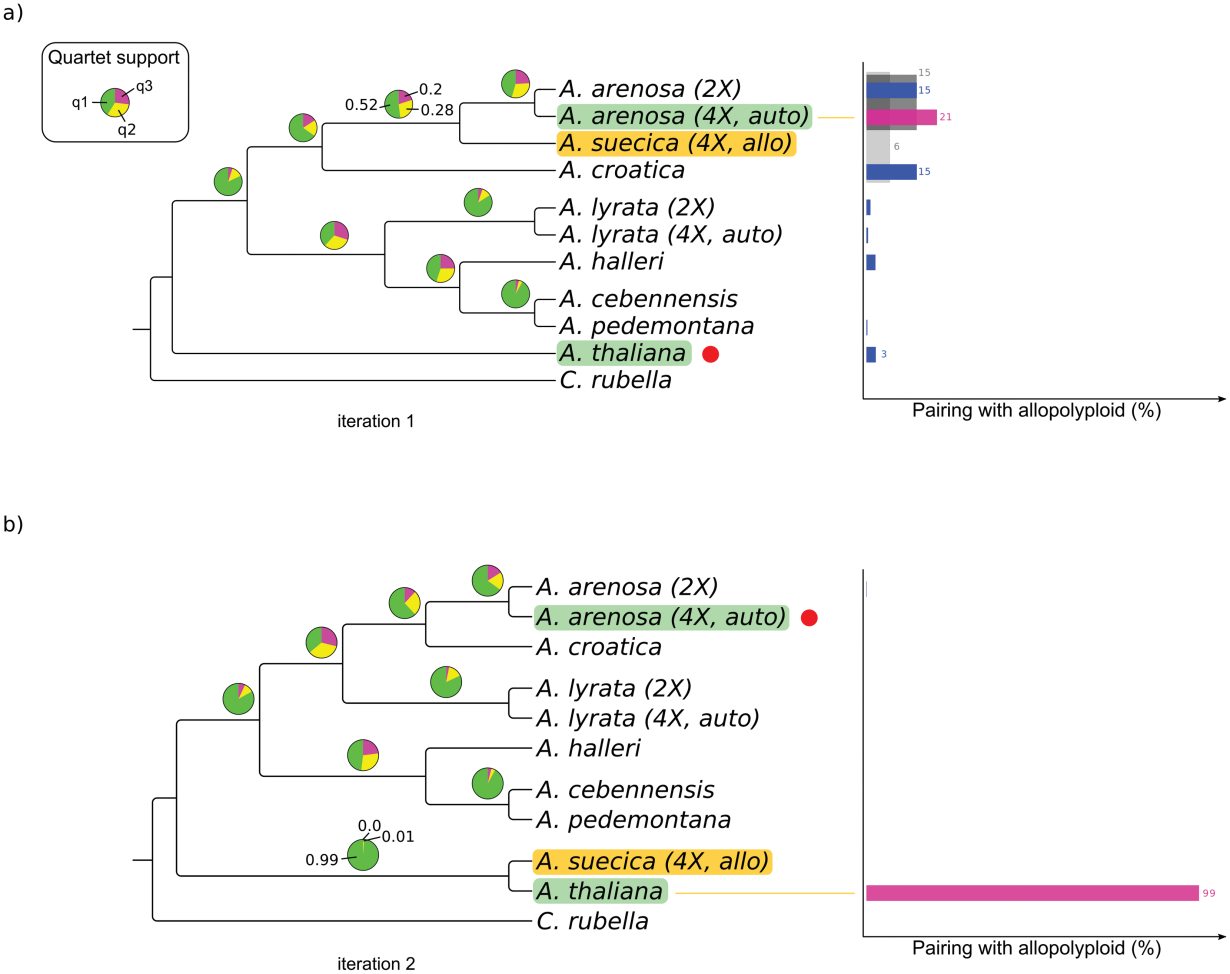
Phylogenetic origins of *A. suecica* confirmed using polarized exonic sequences. Species tree cladograms for the *Brassicaceae* genus, including the allotetraploid *A. suecica*, obtained with ASTRAL based on 752 (*N* = 1) to 2422 (*N* = 2) gene families (exonic sequences only). *Arabidopsis thaliana* and *Arabidopsis arenosa* were identified in previous studies as the parental species of *A. suecica*. (a) Species tree obtained after the first iteration (*N* = 1). *Arabidopsis suecica* sequence polarized using *A. thaliana* as the reference sequence (red dot). *Arabidopsis suecica* pairs with *A. arenosa*. (b) Species tree obtained after the second iteration (*N* = 2). *Arabidopsis arenosa*, having been identified as a parental species during the previous iteration, is now the polarizing reference sequence. *Arabidopsis suecica* pairs with *A. thaliana.* Pie charts show the quartet support for each branch. Precise support values are shown for the focal clade containing the allotetraploid species. Only the *A. suecica* sequence is polarized. Species containing diploid (2×) and autotetraploid (4×, auto) variants are labeled individually. Species with no label are diploid. Barplots on the right-hand side show frequency with which *A. suecica* pairs with other species and is based on phylogenetic analysis of individual gene families using IQ-TREE2. Blocks in gray indicate groupings with more than one species (shown only if support ≥4%, for clarity purposes).

Analysis of *A. suecica*, the second allopolyploid present in the *Arabidopsis* genus, similarly corroborates results obtained in previous studies, providing further evidence that genomic polarization can be used during phylogenetic reconstruction to pinpoint the position of the parental species of an allopolyploid. When *A. thaliana* was employed as the reference sequence, *A. suecica* paired with the two *A. arenosa* species (Fig. 4a). During the second iteration, the tetraploid *A. arenosa* was used as the reference during polarization and *A. suecica* paired with *A. thaliana* (Fig. 4b). Quartet support was very high for the pairing with *A. thaliana* (*q*_*1*_ = 0.99), this in spite of *A. suecica* having a karyotype (2*n* = 4*x* = 26) distinct from that observed in *A. thaliana* (2*n* = 2*x* = 10) or in any of the remaining extant *Arabidopsis* species, the latter all having a base chromosome number *n* = 8 (2*n* = 2*x* = 16 or 2*n* = 4*x* = 32) (see, e.g., Hohmann et al. 2014).

By comparison, the *q*_*1*_ quartet score for the *A. suecica/A. arenosa* clade is somewhat subdued (*q*_*1*_ = 0.52), likely as a reflection of the more complex evolutionary history of the *arenosa* lineage. There is evidence of secondary gene flow and/or ancestral shared polymorphisms between *A. arenosa* and *A. croatica* (Koch and Matschinger 2007; Hohmann et al. 2014; Koch 2019), as well as of rampant gene flow from diploid *A. arenosa* to autotetraploid *A. arenosa* (Jørgensen et al. 2011; Arnold et al. 2015; Monnahan et al. 2019), which would have increased the number of genomic segments with high similarity across species. When phylogenetic inference was carried out separately for each gene family, heightened levels of admixture between species in the *A. arenosa* lineage were therefore expected to increase the probability of *A. suecica* pairing with off-target species. This is visible in the barplot in Fig. 4a, a summary of the results obtained for individual gene families, which shows that *A. suecica* and tetraploid *A. arenosa* were paired 21% of the time, followed by pairings with diploid *A. arenosa*, both *A. arenosa* species (shown in dark gray), and *A. croatica*, each occurring 15% of the time. Despite the spread across multiple topologies, the overall evidence strongly supports pairing with *A. arenosa*, with the highest match occurring for tetraploid *A. arenosa*, in agreement with previous studies that have identified this autopolyploid as the progenitor species, together with *A. thaliana*, most likely to have given rise to *A. suecica* (Jakobsson et al. 2006; Novikova et al. 2016; Koch 2019). Similar results were obtained when the analysis was carried out using only intronic sequences (Supplementary Fig. S3 available on Dryad).

The results described above for *A. kamchatica* and *A. suecica* were based on MSAs generated using short-reads mapped and genotyped against a single reference genome (*A. lyrata*, see Materials and Methods). In order to check for the possible impact of reference genome bias, we re-ran the whole analysis using MSAs generated by employing a different reference genome, *A. halleri*, during read mapping and variant calling. The change of reference genome led to changes in tree topology in one of the case-studies (Supplementary Figs. S4 available on Dryad), namely in the phylogenetic positioning of deep speciation events whose timing is rather uncertain (discussed above). The identity of the parental species of each of the two allopolyploids, as determined using the polarization protocol, was not affected by the choice of reference genome (Supplementary Figs. S4 and S5).

These results provide evidence that the polarization protocol yields accurate results even in the face of major chromosomal rearrangements and significant levels of hybridization and introgression across species. Tests carried out using two different reference genomes during mapping and genotyping suggest that the protocol is also robust against reference genome bias.

## Discussion

### Genomic Polarization Allows for the Simultaneous Segregation and Phasing of Homoeologous Gene Copies

Reconstruction of phylogenies containing allopolyploid species has traditionally required a two-step phasing process (Rothfels 2021) where homoeologous gene copies are first segregated (haplotype phased) before being assigned to one of the subgenomes (phasing of gene copies). Most of the methods currently employed for reconstructing the reticulate history of polyploids require pre-phased data as input, that is, haplotype-phased data where homoeologous gene copies have already been identified and tentatively assigned to one of the subgenomes (even if randomly; Jones et al. 2013; Freyman et al. 2021). In this article, we proposed a different approach that retains the core premise of phasing—to produce two nucleotide sequences that capture the essence of each subgenome—but also greatly simplifies it, reducing the phasing process to a single step. This was accomplished by polarizing the (unphased) allopolyploid consensus sequence directly in the MSA, a process that generates nucleotide sequences that maximize differences between the allopolyploid and a reference sequence. The simple idea behind this strategy is that, if the reference sequence coincides with one of the parental species, the polarized polyploid sequence will closely resemble the second ancestral species. Here, we showed that phasing of an allotetraploid using genomic polarization can be used to pinpoint with high accuracy the two species closest to the polyploid’s parental species (from among those included in the analysis).

Our results show that, in the absence of ILS and quasi-polytomies, 99.95% of all simulation runs correctly identified the position of one of the parental species during the first iteration and that, once the identity of one of the parents is revealed, convergence is assured and the solution found is the true solution. For this particular kind of topologically constrained phylogenies, both parents were correctly identified in all 61,319 cases studied upon convergence of the iterative process. Analysis of synthetic data simulating unconstrained phylogenies with moderate levels of ILS showed that the polarization method can accurately pinpoint both ancestral species of an allotetraploid with 97% accuracy after three to four iterations, while performance with phylogenies containing high levels of ILS yielded an 87% accuracy (Fig. 2).

Arguably, the proposed approach falls short of the type of accuracy typically obtained through two-step phasing. Polarized genomic sequences do not necessarily provide an exact and faithful depiction of each subgenome, even when the reference sequence coincides with one of the allopolyploid’s parental species because polarization entails the scanning of polymorphic sites in the allopolyploid and the “blind” removal of allelic variants present in a reference sequence (as illustrated in Fig. 1). However, our approach does fulfill the purpose of identifying the parental species of an allopolyploid, even in adverse conditions when there is a high degree of incongruence between individual gene trees.

Application of the polarizing protocol to *A. kamchatica* and *A. suecica*, two allotetraploids in the *Arabidopsis* genus whose evolutionary history has been well documented, yielded the same parental pairs as previously identified by other researchers through detailed genetic studies. Both species are neo-allopolyploids estimated to have very recent origin, about 16,000 (*A. suecica;*Novikova et al. 2017) and 20,500 years ago (*A. kamchatica;*Tsuchimatsu et al. 2012). They are also self-fertilizing species exhibiting disomic inheritance (Comai et al. 2003; Tsuchimatsu et al. 2012; Shimizu and Tsuchimatsu 2015; Lloyd and Bomblies 2016; Koch 2019). As a consequence, chromosomes associated with each of their subgenomes tend to be generally homozygous (Paape et al. 2016; Novikova et al. 2017), making these two allopolyploids ideal for testing methods developed for reconstructing the reticulate history of polyploids. The *A. suecica* (2*n* = 4*x* = 26) case study was particularly instructive as its parental species, *A. thaliana* (2*n* = 2*x* = 10) and the tetraploid *A. arenosa* (2*n* = 4*x* = 32), have very distinct karyotypes. This suggests that the polarization protocol can potentially be used even in cases where the allopolyploid’s ancestral species are divergent lineages where at least one of them has undergone major structural chromosomal rearrangements. Phylogenetic analysis of *A. suecica* also allowed us to evaluate whether the presence of species having high genetic similarity to one of the allopolyploid’s parental species—due to gene flow, shared ancestral polymorphisms, or WGD—can hinder the method’s accuracy. The presence of genetic mechanisms inhibiting the diversification of polymorphism patterns across closely related species, while not strong enough to prevent the identification of *A. suecica*’s parental species, could prove a more challenging hurdle in genera distinguished by widespread interspecific hybridization.

### Use of Short-Read Sequencing Data in Polyploid Phylogenomics

Many of the polyploid phylogenetic reconstruction packages available today were originally tested using haplotype-phased data obtained using experimental methods—such as long-read sequencing (Freyman et al. 2021), special purpose primers (Jones et al. 2013; Yan et al. 2022), and flow-sorting technology (Yan et al. 2022)—or a mix of experimentally and computationally phased data (Lautenschlager et al. 2020). One of the motivations behind the development of the polarization protocol was to make reconstruction of polyploid phylogenies using short-read HTS data more accessible. Because our method does not require pre-phasing of individual loci, neither experimental phasing nor computational haplotype phasing needs to be performed. Instead, short reads are used to produce single consensus sequences for each species, including the allotetraploid, which serve as the base upon which MSAs are built for each locus. It is only at this late stage that phasing is performed, using genomic polarization, carrying out the simultaneous segregation and sorting of gene copies in the allopolyploid. The proposed method is suitable for use with both long-read and short-read HTS data, such as whole-genome and -exome capture sequencing data commonly obtained using current Illumina sequencing platforms. Potentially, it can also be used with RAD or RNA sequencing data, although such applications are yet to be tested.

An important question relates to possible adverse effect of using IUPAC consensus sequences (used in the polarization protocol) instead of haplotype-phased sequences (used in two-step phasing) during phylogenetic reconstruction. Studies based on phylogenies containing only diploid species show that phylogenetic reconstruction based on haplotype-phased data provides more accurate estimates of divergence times between species than phylogenies built using IUPAC consensus sequence data, but also that both methods perform equally well when estimating tree topologies (Andermann et al. 2019).

### Polarization Protocol is Built on Top of Standard Pipelines Used in Phylogenetic Analysis of Diploid and Haploid Species

An important advantage of the proposed method is its simplicity, and standard pipelines used in phylogenetic inference of (non-reticulate) diploid and haploid species can be easily modified to incorporate a polarization step. The latter takes an MSA as its input and produces an output which is also an MSA, but in which the allopolyploid sequence, and only the allopolyploid sequence, has been polarized. The only constraint is that the input MSA must contain nucleotide sequences coded using the IUPAC nomenclature and, critically, include all the variants observed for each locus in the polyploid. Such genotype data are now routinely generated when carrying out variant calling on libraries obtained through HTS. The runtime for the polarization step scales linearly with the number of loci included in the analysis, O(n), and it is not expected to be meaningfully affected by the number of species present in the MSA, as only the allotetraploid is polarized. In the analysis of the *Arabidopsis* phylogenies carried out in this study, it took 1 hour to process 24k genes on a 64-bit Linux cluster using a single Intel Xeon E5-2630 V4 processor running at 2.2 GHz and 6.4 GB RAM. This step can be further sped-up using parallelization.

### Genomic Polarization Protocol: When to Use It

The polarization protocol requires no prior knowledge about the likely origins of the allotetraploid nor for its diploid progenitors to be extant or sampled, a flexibility shared with AlloppNET and homologizer (Oxelman et al. 2017; Freyman et al. 2021). In its current implementation, the polarization protocol is limited to the analysis of allotetraploid species. Similar limitations exist for AlloPPnet (Jones et al. 2013; Jones 2017), while AllCoPol, MPAllopp, and homologizer can all process higher ploidy species, with some of them further having the ability to process tetraploids and higher ploidy allopolyploids whose parental species are themselves polyploids (Lautenschlager et al. 2020; Freyman et al. 2021; Yan et al. 2022). Currently available methods are computationally demanding, which in some cases may limit the number of species (e.g., AlloPPnet (Rothfels et al. 2017); homologizer (Freyman et al. 2021)) or loci (e.g., AlloPPnet (Yan et al. 2022); AllCoPol (Lautenschlager et al. 2020); see also Oberprieler et al. 2017) that can be included in the analysis in practice. Such limitations do not exist when using genomic polarization.

Like all the methods mentioned above, the polarization approach is better suited for the analysis of neopolyploids, that is, allopolyploids whose constituent subgenomes remain distinct and are therefore only at the beginning of the diploidization process, a series of large-scale structural rearrangements, both within and across subgenomes, set in motion in the aftermath of polyploidization (Soltis and Soltis 1999; Dodsworth et al. 2016). More specialized tools have been proposed for the phylogenetic analysis of mesopolyploids, that is, allopolyploid species that have undergone some level of genome reshuffling and gene fractionation across their subgenomes (Hénocq et al. 2020).

A precondition for using the polarization protocol is that the taxonomic group of interest must contain extant diploid and/or autopolyploid species, as these will be used to build a bifurcating tree that defines the phylogenetic space in which the search for the allopolyploid parental species takes place. This condition is easily satisfied in many plant genera, such as *Betula* (Wang et al. 2021) and *Coffea* (Lashermes et al. 1999), and also in several metazoan families (Schmid et al. 2015), often ectothermic (Mable et al. 2011), containing (or suspected to contain) extant functional allopolyploids, such as leptodactylid frogs (Faivovich et al. 2012) and Palearctic green toads (Betto-Colliard et al. 2018; Dufresnes et al. 2019). If any allopolyploids, besides the focal allotetraploid, are included in the analysis—for instance, because the focal allopolyploid is suspected to be the hybrid offspring of two allopolyploid species—then they should be represented by their subgenomes and must therefore be phased beforehand using the protocol proposed in this paper. In its current form, the protocol is of limited use in the study of groups where most extant lineages are polyploid, such as the *Xenopus* genus of clawed frogs (Evans 2008) or the Salmonidae, Callichthyidae, and Catostomidae fish families (Braasch and Postlethwait 2012).

### Required Number of Individuals per Species

Phylogenetic reconstruction using the genomic polarization method described in this article assumes that each species is represented by one single individual, thereby disregarding the breadth of allelic variation within each species. There is evidence that sampling multiple individuals per species can help minimize bias and errors during phylogenetic inference incurred due to ILS (Maddison and Knowles 2006; McCormack et al. 2009; Heled and Drummond 2010). Some of these studies further suggest that higher accuracy can often be obtained by increasing the number of samples rather than by increasing the number of genes included in the analysis (Maddison and Knowles 2006; McCormack et al. 2009). These results, however, seem to be dependent on the actual methodology used. In a recent study carried out using ASTRAL, the tree reconstruction method we employ in this article, better results were obtained by sampling more genes than by sampling more individuals, likely because simulations carried out using ASTRAL included gene sets with up to one thousand loci, while previous studies included at most 50 genes (Rabiee et al. 2019).

### Availability of a Reference Genome

In the pipeline used in this article, short reads were used to build a consensus sequence for each of the species included in the *Arabidopsis* phylogeny, and these were subsequently used to create an MSA for each locus. The *A. lyrata* reference genome was used for read mapping, variant calling, and building the consensus sequence for all the species included in the analysis. As only about 0.2% of all extant plant and animal species have their genomes sequenced and assembled (Hotaling et al. 2021; Marks et al. 2021), the question may arise as to whether the small number of potentially available reference genomes (~800 plant species; ~3280 animal species) might limit the applicability of the polarization protocol. There are currently several large-scale projects aimed at vastly expanding the number of available animal, fungus, and plant genomes (reviewed in Lewin et al. 2018). Additionally, there are several pipelines available for carrying out genotyping in the absence of a reference genome, including also in polyploid species, which generate a *de novo* reference based on the available reads (Catchen et al. 2011; Lu et al. 2013; Melo et al. 2016; Qi et al. 2018).

It has been noted that aligning short reads against a single reference genome often introduces systematic biases in downstream analyses, such as during variant calling, and that different results might be obtained depending on the particular genome used for mapping (Pool et al. 2010; Sousa and Hey 2013; Bertels et al. 2014; Gopalakrishnan et al. 2017; Hurgobin and Edwards 2017). Reference genomes are often obtained from a single inbred, highly homozygous individual, and even when this is not the case, they are unlikely to capture the entire genetic diversity of a given species. Additionally, when mapping reads from more distant taxa, the increased genomic divergence between the reference genome and the focal species may negatively impact the ability of the mapper to successfully and unambiguously align reads to the reference. In some cases, reference genome bias might be strong enough to affect the topology of the inferred phylogeny (Bertels et al. 2014; Gopalakrishnan et al. 2017). In order to account for the possibility of reference genome bias, we employed several complementary strategies. Whenever possible, we selected accessions with high-coverage sequencing (Supplementary Table S2 available on Dryad), as it has been shown that reference bias can be alleviated by increasing depth and coverage during sequencing (Pool et al. 2012). We also restricted the analysis to single-copy orthogroups in order to minimize false positives during variant calling stemming from (mis-)mapping to paralogous genes. Additionally, we filtered out sites in the MSAs for which most species were masked, as such sites are often associated to low quality, and therefore ambiguous, variant calls. Finally, we re-ran the whole analysis using a reference genome based on a different *Arabidopsis* species, *A. halleri*, during mapping and variant calling. While we occasionally observed slightly different tree topologies depending on which reference genome was used, the phylogenetic positioning of the allopolyploid’s parental species was not affected by the choice of reference genome in none of the cases studied (compare Fig. 3 and Supplementary Fig. S4, and Fig. 4 and Supplementary Fig. S5).

### Future Directions

The polarization protocol can potentially be used to identify genomic regions biased toward one of the parental species (i.e., cases where one of the subgenomes is overrepresented) and evaluate whether such regions are few and far in between or, alternatively, whether they extend over large genomic tracks. In allopolyploids exhibiting disomic inheritance patterns, such as *A. suecica* and *A. kamchatica*, we would expect very few instances of genomic bias as there is little or no recombination between subgenomes. Inversely, if there is homoeologous recombination or homoeologous replacement, we would expect to observe genomic bias in regions where, for instance, a particular allele has attained fixation across subgenomes. This approach could also be used to investigate species where the likelihood of multivalent pairing, and thus of homoeologous recombination, varies among different chromosomes due to variations in the degree of homoeologous synteny, a process that has been observed in the *Brassica napus* Stellar cultivar, an allotetraploid, and also in newly resynthesized *B. napus* lines (Xiong et al. 2021). In order to distinguish between spurious and true instances of genomic bias, both applications would generally require analysis of a large number of individuals belonging to the allopolyploid species of interest.

Other possible applications include the study of polyploids that hybridize with species other than any of the parental species, as well as of species, such as the allotetraploid peanut plant *Arachis hypogaea*, where trans-introgression between parental species and the allopolyploid can potentially lead to interspecific gene flow from one of the parental species to the subgenome associated with the second parental species in the polyploid (Leal-Bertioli et al. 2018). In both situations, increased levels of genomic bias could help identify genomic regions where specific alleles are favored, possibly as a consequence of natural selection or local adaptation.

## Supplementary Material

Data available from the Dryad Digital Repository: http://dx.doi.org/10.5281/zenodo.5826401.

## Data Availability

Supplementary materials, scripts, and data available from the Dryad Digital Repository: https://doi.org/10.5061/dryad.dfn2z353j and the GitHub repository https://github.com/LLN273/Genomic_polarization_allotetraploids. The WGS data underlying this article are available in the European Nucleotide Archive (ENA) at www.ebi.ac.uk and can be accessed with the PRJNA284572 (https://www.ebi.ac.uk/ena/browser/view/PRJNA284572), and PRJNA273563 (https://www.ebi.ac.uk/ena/browser/view/PRJNA273563), accession codes.
